# Nipah Virus Encephalitis: Pathogenetic Aspects and Current Therapeutic Strategies

**DOI:** 10.3390/pathogens15040443

**Published:** 2026-04-20

**Authors:** Gaetano Scotto, Vincenzina Fazio, Ali Muhammed Moula Ali, Sri Charan Bindu Bavisetty, Alessia Franza, Salvatore Massa

**Affiliations:** 1 Department of Biomedical Sciences, University of Foggia, 71121 Foggia, Italy; 2 Department of Life Sciences, School of Science, Gandhi Institute of Technology and Management (GITAM) University, Hyderabad 502329, India; amoulaal@gitam.edu; 3 Faculty of Food Industry, King Mongkut’s Institute of Technology Ladkrabang, Bangkok 10520, Thailand; sricharanbindu.ba@kmitl.ac.th; 4 Department of Agricultural, Food and Environmental Sciences, University of Foggia, 71121 Foggia, Italy; alessia_franza.542647@unifg.it

**Keywords:** Nipah virus, encephalitis, respiratory disease, neuropathogenesis, pulmonary involvement, monoclonal antibodies, vaccine development, zoonotic virus

## Abstract

Nipah virus (NiV) is a highly pathogenic zoonotic paramyxovirus responsible for sporadic outbreaks of severe disease with high case fatality rates in South and Southeast Asia. Human infection occurs through spillover from natural reservoirs, primarily fruit bats, or via human-to-human transmission, and is characterized by a broad clinical spectrum ranging from asymptomatic infection to acute respiratory disease and fatal encephalitis. Following entry via ephrin-B2 and ephrin-B3 receptors, NiV exhibits marked endothelial and neuronal tropism, leading to systemic vasculitis, disruption of the blood–brain barrier, and direct infection of the central nervous system. Disease progression is driven by a complex interplay between viral replication strategies and host immune responses. NiV effectively counteracts innate immunity through multiple viral proteins that inhibit interferon signaling, while simultaneously inducing dysregulated inflammatory responses that contribute to tissue damage and multi-organ failure. Neurological involvement represents the most severe manifestation, often resulting in acute or relapsing encephalitis with long-term sequelae among survivors. Despite the severity of the disease, no licensed antiviral therapies or human vaccines are currently available. Therapeutic development has focused on neutralizing monoclonal antibodies targeting viral glycoproteins and small-molecule antivirals that inhibit viral RNA synthesis, both of which show promising results in preclinical models, but remain limited by timing and translational challenges. In parallel, several vaccine platforms—including viral vectors, mRNA-based constructs, and recombinant protein subunits—have advanced to early-phase clinical trials, demonstrating encouraging immunogenicity. Beyond biomedical interventions, effective outbreak containment relies on integrated public health strategies. The “Kerala model” highlights the importance of rapid case identification, isolation, contact tracing, and community engagement within a One Health framework to mitigate transmission and reduce mortality. This review synthesizes the current knowledge on NiV pathogenesis, immune evasion, clinical manifestations, and emerging therapeutic and vaccine strategies, while highlighting critical gaps and future directions for improving the preparedness and response to this high-consequence emerging pathogen.

## 1. Introduction

In recent decades, the emergence and re-emergence of viral diseases has become a major concern for global public health. This trend is driven by a complex interplay of biological, environmental, and anthropogenic factors. Approximately 60–75% of newly identified infectious diseases are zoonotic in origin, resulting from cross-species transmission events—so-called “spillover”—from animal reservoirs to humans [1,2]. Concurrently, globalization has dramatically accelerated the potential for the rapid geographical spread of novel pathogens, while climate change is altering the ecological niches of wildlife species, thereby increasing the frequency of human–animal interfaces [3]. At the molecular level, RNA viruses are particularly prone to evolutionary diversification owing to the high error rate of their RNA-dependent RNA polymerase, enabling rapid antigenic variation and immune evasion. Taken together, these factors have substantially heightened the risk of zoonotic spillover events and the potential emergence of novel epidemic or pandemic threats [4].

Within this landscape, the family *Paramyxoviridae* has attracted increasing scientific attention. More recently, this family of RNA viruses has been expanded to include the genus *Henipavirus*, which encompasses viruses capable of causing severe zoonotic diseases with high case fatality rates. Among these, the Nipah virus (NiV) and the Hendra virus (HeV) have emerged as particular concerns due to their demonstrated ability to cross species barriers and their significant pathogenic potential in humans [1].

Nipah virus (NiV) was first identified during an outbreak in Malaysia and Singapore between 1998 and 1999 and named after the village of Sungai Nipah in the Malaysian state of Negeri Sembilan, where it was first isolated from patient samples presenting with encephalitis e [5,6]. Fruit bats of the genus *Pteropus* have been identified as the natural reservoir hosts [7]. Zoonotic transmission to humans occurs primarily through direct contact with infected bats or, more commonly in endemic regions such as Bangladesh, through the consumption of raw date palm sap contaminated with bat excreta [6,7]. Human-to-human transmission has also been well documented, particularly in healthcare settings, further amplifying the public health burden associated with NiV outbreaks [5,8].

Phylogenetic analyses have revealed two genetically distinct lineages of NiV—the Malaysian (NiV-M) and Bangladeshi (NiV-B) strains—with the latter demonstrating a greater genetic heterogeneity and, importantly, a higher pathogenicity and transmissibility compared to the Malaysian strain [9,10,11,12]. These findings highlight the ongoing evolutionary dynamics of NiV and underscore the importance of continuous genomic surveillance [7].

Clinically, NiV infection presents with a spectrum of manifestations. The initial symptoms are typically non-specific, including a fever, a headache, myalgia, a cough, and a sore throat. However, the disease may progress rapidly to severe respiratory distress and, most critically, to encephalitis characterized by dizziness, disorientation, confusion, and seizures, which can culminate in a coma within 24–48 h [6,13]. Case fatality rates have been reported to range between 40% and 95%, underscoring the extreme severity of NiV-associated encephalitis [5,12,14].

In recognition of its high lethality, zoonotic transmission, and characteristic epidemic/pandemic potential, the World Health Organization [15] designated NiV as a priority pathogen, calling for intensified research on its epidemiology, its pathogenesis, and the development of countermeasures [2,6]. Accordingly, NiV is classified as a Biosafety Level 4 (BSL-4) agent, reflecting the extreme biosafety requirements associated with its handling [5,16,17,18]. Hence, the practical limitations for conducting functional genomics studies at high levels of containment become apparent [19].

Despite decades of research, no specific antiviral therapies or licensed vaccines for NiV are currently available; management of infected individuals relies on supportive care alone [18,20]. The absence of effective therapeutic options, combined with the unpredictability of zoonotic spillover events, represents a critical gap in global infectious disease preparedness.

The present review aims to provide a comprehensive analysis of NiV encephalitis, focusing on two central domains. First, the pathogenetic mechanisms underlying NiV neurotropism are examined in detail, with particular emphasis on the role of ephrin-B2/ephrin-B3 receptors, vascular endothelial involvement, blood–brain barrier disruption, and the routes of viral entry into the central nervous system [18]. Second, the current therapeutic landscape is critically appraised, encompassing recent advances in immunotherapy—including the use of humanized monoclonal antibodies—as well as an evaluation of antiviral agents, such as remdesivir and novel polymerase inhibitors, and the status of ongoing vaccine development, notably the ChAdOx1-NipahB candidate currently in Phase I clinical trials [20]. By integrating pathogenetic and therapeutic perspectives, this review seeks to identify priority areas for future research and to contextualize current efforts within the broader framework of emerging infectious disease preparedness.

## 2. Taxonomy

**Figure 1:**
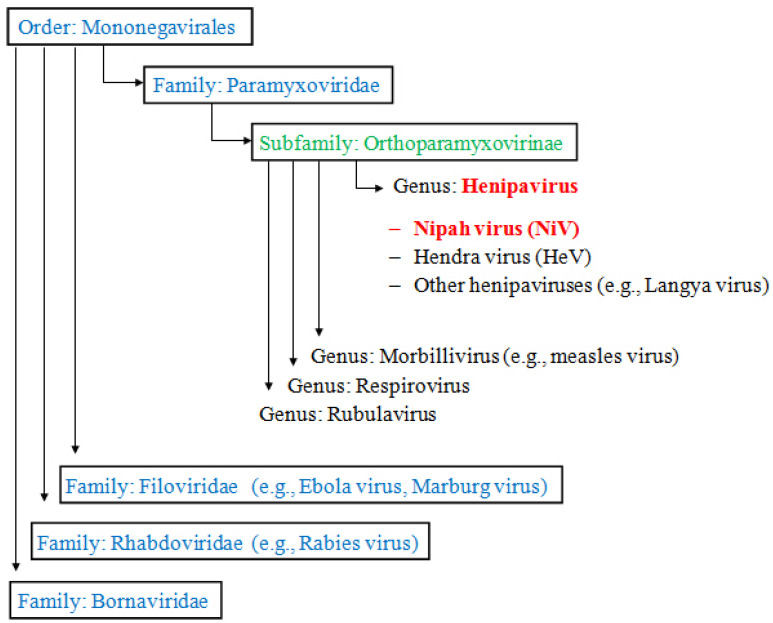
Phylogenetic tree of representative viruses in the order *Mononegavirales*. This unrooted tree was constructed using conserved polymerase protein sequences. The order includes medically and veterinary important families: *Paramyxoviridae*, *Filoviridae*, *Rhabdoviridae*, and *Bornaviridae* (see main text). Subfamilies are shown in green, genera in red, and unassigned paramyxoviruses are boxed. The genus *Henipavirus* (including Nipah virus [NiV] and Hendra virus [HeV]) is highlighted within *Paramyxoviridae*. Other henipaviruses such as Cedar henipavirus, Ghanaian bat henipavirus, Mojang henipavirus, Langya henipavirus, and Camp Hill virus (recently identified) are not shown (adapted from [30]).

The Nipah virus (NiV; *Henipavirus nipahense*) belongs to the order Mononegavirales, a large group of enveloped viruses characterized by non-segmented, negative-sense single-stranded RNA genomes. Members of this order share several fundamental features, including a conserved genomic organization, a helical nucleocapsid structure, and a replication strategy mediated by a viral RNA-dependent RNA polymerase. These similarities reflect ancient evolutionary relationships among the viruses included in this order. The order *Mononegavirales* comprises several viral families of major medical and veterinary importance, including *Paramyxoviridae*, *Filoviridae*, *Rhabdoviridae*, and *Bornaviridae* [6,21] (Figure 1).

The family *Paramyxoviridae* is a diverse group of viruses infecting humans and animals that are responsible for a wide spectrum of diseases ranging from mild respiratory infections to severe systemic illnesses. Historically, this family includes well-known human pathogens such as the measles virus and the mumps virus, as well as respiratory syncytial virus (RSV), parainfluenza viruses, and metapneumoviruses, which have caused substantial morbidity worldwide [6,22]. Although the lethal impact of many human paramyxoviruses has been dramatically reduced through vaccination, animal pathogens in this family have caused devastating effects on livestock and poultry industries globally, highlighting the broad veterinary relevance of this group [6].

Within the family *Paramyxoviridae*, NiV belongs to the genus *Henipavirus*, whose prototypical members are Hendra virus (HeV) and Nipah virus (NiV). The *Henipavirus* genus also includes other related viruses such as Cedar henipavirus (CedV) [23], Ghanaian bat henipavirus (GhV) [24], Mojang henipavirus (MojV) [25], Langya henipavirus (LayV) [26], and the recently identified Camp Hill virus (CHV) in North America [27]. To date, epidemiological studies have not documented human infections caused by CHV; however, CHV shares a close genetic relationship with LayV, which has demonstrated zoonotic transmission from shrews to humans in China. The discovery of CHV represents the first report of a henipavirus in North America, substantially expanding the recognized geographic range of this viral genus [27].

Henipaviruses are distinguished by their broad host range and pronounced zoonotic potential. Their natural reservoirs are fruit bats of the genus *Pteropus*, from which spillover transmission may occur to a variety of mammals, including pigs, horses, and humans [6,28]. At the genomic level, NiV exhibits a close evolutionary relationship with HeV, sharing approximately 68–92% homology in coding regions and 40–67% in non-translated regions [29].

## 3. Viral Structure and Genome Organization

**Figure 2:**
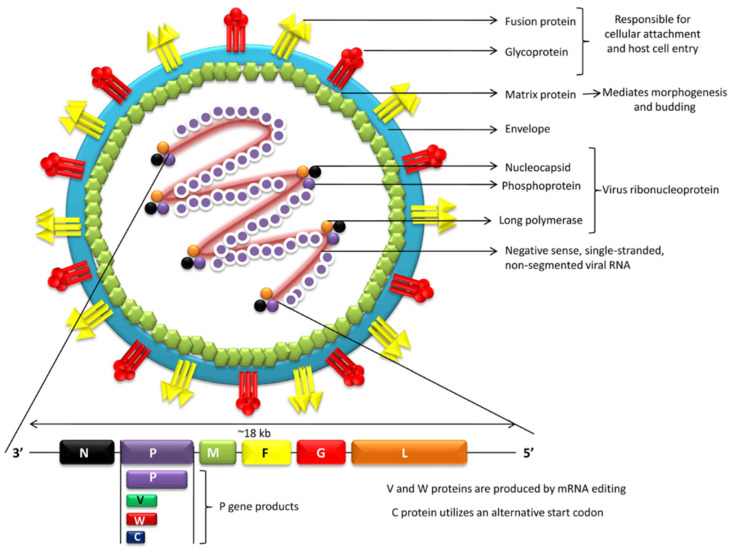
Schematic representation of Nipah virus (NiV) structure and genome organization.

NiV particles exhibit a varied morphology, appearing as spherical or filamentous structures [31]. The outer viral surface is characterized by a lipid bilayer envelope that is derived from the plasma membrane of the host cell in which the virus is grown. Embedded into the envelope are tetrameric attachment glycoproteins (G) and trimeric fusion proteins (F), which extend approximately 5–8 nm from the surface of the membrane and sometimes aggregate into distinct clusters [32,33] (Figure 2). G and F glycoproteins represent the main antigens against which NiV nAbs are produced, and therefore, they are particularly studied from an immunological point of view as potential vaccine candidates [34,35]. The inner layer is formed by matrix proteins (M) that make up the viral capsid. Inside the capsid, there is a single non-segmented, negative-stranded (-ve) ribonucleic acid (RNA) genome [22] attached to nucleocapsid viral proteins (N), phosphoproteins (P), and a large (L) RNA polymerase enzyme (RNA-dependent RNA polymerase (RdRP)) [32] (Figure 2). The NiV genome has been fully sequenced and consists of 18,246 base pairs in length and six genes (N, P, M, F, G, L), each encoding one of the six major structural proteins, as illustrated in Figure 2 [29,36,37]. Nucleoprotein N binds tightly to the RNA genome. The F and G proteins insert within the host-derived cell membrane and allow binding and entry into the target cell. The P gene produces multiple proteins: P, the unmodified protein, acts as a cofactor for the viral polymerase complex; V and W are generated through an RNA-editing process (insertion of non-templated guanine nucleotides); and C is translated from an alternative start codon. These proteins modulate interferon responses and contribute to the virus’s pathogenicity [38,39,40].

The genome of NiV has a size of approximately 18 kb, which is considerably larger than the relatively consistent size range of 15.1–15.9 kb observed in other members of the same family [36,41]. This additional length in the genome is primarily located in long untranslated regions, particularly at the 3′ end of five out of the six transcription units [42]. NiV particles have a larger diameter (500 nm) compared to other paramyxoviruses (150–400 nm), with considerable size variations (180–1900 nm), although they exhibit similar morphological characteristics [43]. Using negative-contrast electron microscopy, it is possible to differentiate NiV from HeV based on the surface projections: HeV displays a double-fringed surface projection, whereas NiV typically exhibits a single fringe [29,43]. The two lineages of NiV—the Malaysian (NiV-MY) and Bangladeshi (NiV-BD) strains—differ slightly in genome size, with the Malaysian variant containing 18,246 nucleotides compared to 18,252 nucleotides in the Bangladesh variant [44]. While both variants are functionally similar, they exhibit notable differences in their disease-causing potential and transmission characteristics [9]. Research using African green monkey models revealed that the Bangladesh strain demonstrated greater virulence compared to the Malaysian variant [9,11,45]. Additionally, ferret studies indicated that Bangladesh strain infections result in enhanced viral shedding through oral routes, more rapid disease progression, and increased viral replication within respiratory tissues [35].

These biological differences account for the distinct clinical patterns observed across geographic regions. Infections caused by Bangladesh and Indian strains typically feature reduced incubation times, more pronounced respiratory manifestations, enhanced person-to-person transmission, and elevated mortality rates. In contrast, Malaysian strain infections predominantly manifest as neurological diseases with brain involvement [4].

## 4. Epidemiology

Nipah virus outbreaks have been primarily observed in South and Southeast Asia, in countries that include Malaysia, Singapore, Bangladesh, and India [46,47,48]. Since its first identification in 1998–1999, the epidemiological pattern has evolved significantly, with distinct outbreak profiles emerging across different endemic regions.

### 4.1. Natural Reservoirs

Fruit bats of the genus *Pteropus* serve as the natural reservoir, harboring the virus asymptomatically and disseminating it through saliva, urine, and feces [28,49]. A critical feature of this reservoir system is that infected bats exhibit viral shedding without seroconversion providing lasting immunity; consequently, reinfection leads to persistent viral circulation within bat populations [28,50]. The distribution of *Pteropus* species—including *P. vampyrus*, *P. hypomelanus*, *P. lylei*, and *P. giganteus*—overlaps all documented outbreak locations across Africa, South and Southeast Asia, Australia, and numerous South Pacific islands [51,52].

### 4.2. Transmission Pathways

**Figure 3:**
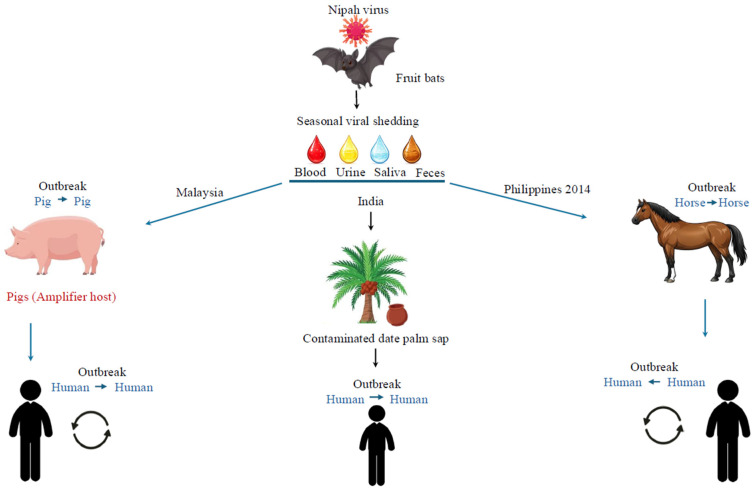
Transmission pathways and outbreak dynamics of Nipah virus (NiV). Schematic representation of the ecological cycle and principal transmission routes of NiV. Fruit bats of the genus *Pteropus* serve as the natural reservoir and intermittently shed the virus through blood, urine, saliva, and feces, leading to environmental contamination of fruit and raw date palm sap. In Malaysia, pigs act as amplifier hosts, supporting pig-to-pig transmission and subsequent zoonotic transmission to humans. In India and Bangladesh, outbreaks are more commonly associated with direct bat-to-human spillover through consumption of contaminated date palm sap, followed by secondary human-to-human transmission. In the 2014 Philippines outbreak, horses served as amplification hosts, with transmission occurring through consumption of contaminated horse meat. The diagram illustrates the dual epidemiological patterns of NiV infection: amplification through an intermediate host and direct zoonotic spillover with subsequent person-to-person spread. Arrows indicate transmission pathways between hosts and humans, and colors distinguish ecological compartment.

Zoonotic spillover to humans occurs through multiple pathways. Bat-to-human transmission represents the predominant route in Bangladesh and India, resulting from consumption of raw date palm sap or fruit contaminated with bat excreta [52,53,54] (Figure 3). This local practice, which involves sap collection in open containers during winter months (December–April), coincides with periods of increased bat activity near human settlements, thereby creating a high contamination risk [55,56].

Intermediate amplification hosts have played critical roles in certain outbreak contexts. The Malaysia–Singapore outbreak (1998–1999) exemplifies this dynamic, wherein intensive pig farming facilitated rapid viral amplification among naïve swine populations, which subsequently transmitted the virus to farm workers through contact with infected bodily fluids [57,58]. The outbreak epicenter in Ipoh village represented a critical convergence point of commercial fruit production, pig farming, and bat circulation, enabling efficient bat–pig–human transmission [29]. This outbreak resulted in 265–276 cases with 105–106 deaths (case fatality rate of approximately 40%), with farmers and abattoir workers bearing the greatest burden [29]. In the 2014 Philippines outbreak, horses served as amplification hosts, with transmission occurring through consumption of contaminated horse meat [59]. Additionally, domestic and peridomestic animals, including cats, dogs, and cattle, have demonstrated seropositivity during outbreak investigations, indicating a broader spillover potential beyond recognized amplification hosts [60].

Human-to-human transmission has distinguished recent outbreaks from earlier epidemic events. Secondary transmission through close contact with infected individuals, particularly during caregiving activities, has been documented extensively in Bangladesh and India [61,62]. Although the infectivity of henipaviruses is lower compared to that of the recent coronavirus (COVID-19) pandemic, their mortality rate is substantially higher, exceeding 60% for henipaviruses compared to a mortality rate of less than 1% for COVID-19 globally [44]. The transmission mechanisms include both respiratory and direct contact routes. Patients with respiratory involvement can generate infectious aerosols during coughing or sneezing, facilitating airborne transmission in close-contact settings, while direct contact with bodily fluids enables transmission through mucous membrane exposure or percutaneous inoculation [44].

A crucial epidemiological parameter for assessing the pandemic potential is the basic reproductive number (R_0_). For human-to-human transmission of NiV, R_0_ has been estimated at approximately 0.5 in community settings, suggesting that sustained transmission chains are unlikely under normal circumstances [62,63]. To contextualize this finding, COVID-19 exhibited R_0_ values of 2–3 during the early pandemic phase, explaining its capacity for rapid global spread, despite substantially lower mortality rates. However, super-spreading events—wherein single infected individuals transmit to unusually large numbers of secondary cases—have been observed for NiV and are influenced by factors that include the viral load, the symptomatic presentation, and culturally specific practices around illness and death. The potential for viral adaptation to enhance the human-to-human transmissibility remains a significant concern, as relatively minor genetic changes could potentially elevate R_0_ above the epidemic threshold of 1.0, thereby enabling sustained transmission chains and larger outbreaks [62,63].

Nosocomial transmission represents a critical amplification pathway that has been prominently featured in recent outbreaks. Healthcare settings have documented extensive transmission among patients, healthcare workers, and family members, particularly in resource-limited settings with inadequate infection control infrastructure [62,64]. The 2001 Siliguri outbreak in India, which involved 66 cases with a 74% mortality, exemplified the risk of efficient hospital-based spread [64]. More recently, Kerala experienced repeated healthcare-associated clusters in 2018, 2019, 2021, and 2023, with the 2018 event achieving a very high 73.9% mortality rate (17 deaths among 23 confirmed cases), underscoring the persistent threat posed by nosocomial transmission [65,66]. The major documented outbreaks reported to date are summarized in Table 1.

### 4.3. Geographical Distribution and Outbreak Patterns

Bangladesh has experienced near-annual outbreaks since 2001, concentrated in the central-northwestern region known as the “Nipah belt” [80,81]. These outbreaks exhibit several distinctive epidemiological features: a marked seasonality occurring between December and April, direct bat-to-human transmission without a requirement for intermediate amplification hosts, high rates of human-to-human spread, prominent respiratory involvement, and a median mortality rate of 75% [56,82,83]. This pattern contrasts sharply with the Malaysian strain’s pig-mediated, non-seasonal transmission profile that lacked sustained human-to-human spread.

India has reported outbreaks in West Bengal (2001, 2007) and Kerala (2018, 2019, 2021, 2023, 2024), with epidemiological characteristics similar to those observed in Bangladesh, including human-to-human transmission and respiratory presentations [66,84]. Kerala has emerged as a persistent endemic hotspot characterized by repeated healthcare-associated clusters.

The potential for geographical expansion beyond current endemic areas warrants continuous monitoring. Serological surveys have detected NiV antibodies in bat populations across Thailand, Cambodia, Indonesia, and the Philippines, suggesting a wider viral distribution than outbreak surveillance data would indicate [85]. In Australia, formal risk assessments indicate a low probability of NiV establishment in native Pteropus populations, though rapid ecological changes driven by anthropogenic land-use patterns could alter the transmission dynamics [86,87]. In Africa, frugivorous bats of the genus Eidolon have tested seropositive for henipavirus antibodies, raising concerns about unrecognized viral circulation in regions that currently lack systematic human outbreak surveillance [1].

### 4.4. Ecological and Anthropogenic Drivers

Multiple environmental and anthropogenic factors contribute to an elevated spillover risk. Habitat destruction and land-use changes have disrupted traditional bat foraging patterns, thereby increasing the frequency of bat–human interfaces as Pteropus populations increasingly utilize agricultural areas and human settlements for feeding and roosting [62]. Climate-driven alterations in bat migratory patterns further compound these effects by expanding their geographical ranges while potentially stressing populations in ways that may enhance viral shedding [88]. The establishment of large-scale livestock operations in regions overlapping with flying fox habitats creates ideal conditions for viral amplification and subsequent human exposure, a dynamic exemplified by the Malaysian outbreak experience [62].

The molecular basis for NiV’s remarkably broad host tropism lies partly in the evolutionary conservation of ephrin-B2 and ephrin-B3 receptors across diverse mammalian species [89,90]. Experimental evidence suggests that relatively few mutations are required to enhance the henipavirus transmission efficiency in novel host species, thereby emphasizing the evolutionary plasticity of these viruses and their potential for rapid adaptation to different cellular environments [89].

Socioeconomic factors—including a limited healthcare infrastructure, inadequate disease surveillance systems, and cultural practices surrounding caregiving and burial rituals—can further facilitate viral spread once spillover events have occurred [44]. Additionally, ongoing deforestation for agricultural expansion creates self-reinforcing feedback loops that simultaneously intensify the spillover risk while constraining the resources available for prevention and outbreak responses. This complex interplay between natural reservoir dynamics, intermediate host amplification, ecological disruption, and anthropogenic pressures underscores the fundamentally multidimensional nature of NiV epidemiology and highlights the critical need for integrated One Health surveillance and intervention strategies that span human, animal, and environmental health sectors.

## 5. Pathogenesis, Immune Evasion, Encephalitis, and Clinical Manifestations of Nipah Virus Infection

### 5.1. Pathogenesis of Nipah Virus Infection

**Figure 4:**
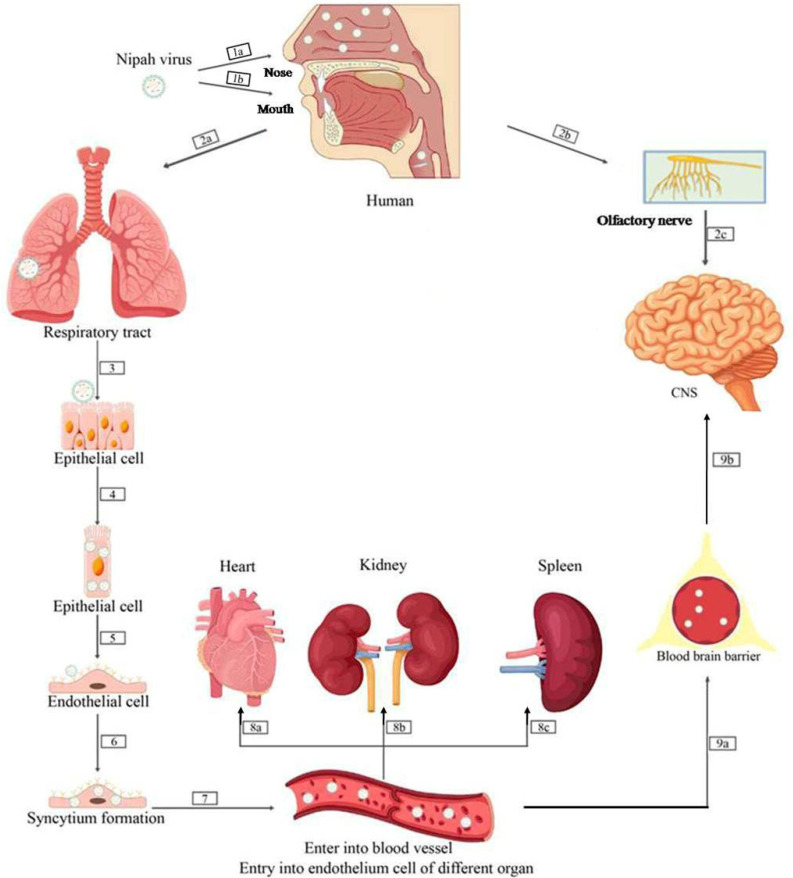
Pathogenesis and progression of Nipah virus (NiV) infection. (1a–b) NiV enters the host through the respiratory mucosa (inhalation of infected droplets) or the gastrointestinal tract (ingestion of contaminated food). Regardless of the route of exposure, the virus localizes to the respiratory tract, where primary infection is established. (2a–3–4) NiV infects epithelial cells of the upper and lower respiratory tract, where initial viral replication occurs (synthesis of structural and nonstructural proteins, replication of the NiV genome, and assembly of viral particles). (5) NiV uses F and G proteins to attach to ephrin-B2/B3 receptors, leading to endothelial infection. (6) Endothelial infection induces syncytium formation, inflammation, and vasculitis, with disruption of vascular integrity. (7) The virus enters the bloodstream (viremia) and disseminates systemically. (7, 8a–8b–8c) Hematogenous spread results in infection of various organs, including the lungs, heart, kidneys, and spleen, potentially leading to multiple organ dysfunction syndrome (MODS). (9a–9b) Infection of cerebral microvascular endothelial cells leads to blood–brain barrier (BBB) disruption. NiV enters the central nervous system, causing encephalitis characterized by vascular injury and neuronal infection. In parallel, NiV may directly access the central nervous system via retrograde transport along the olfactory nerve (2b–c), bypassing the blood–brain barrier and providing an additional route of neuroinvasion (adapted from [6]).

Nipah virus (NiV) infection follows a complex, multistep pathogenesis that includes viral entry, replication, systemic dissemination, and multi-organ involvement [91]. The overall progression is illustrated in Figure 4, which guides the reader through each stage from initial exposure to central nervous system (CNS) invasion.

NiV gains entry into the host primarily via the respiratory mucosa through inhalation of infectious droplets or through the gastrointestinal tract following ingestion of contaminated food [6]. Experimentally inoculated animals have demonstrated that primary replication of the virus occurs in the epithelial cells of the upper and lower respiratory tract, regardless of the route of inoculation [10,39,92] These epithelial surfaces are critical sites for viral amplification and shedding, supporting transmission to susceptible hosts [93].

Viral entry is mediated by the interaction of the G attachment glycoprotein (Figure 2) with the host ephrin-B2 (EFNB2) and ephrin-B3 (EFNB3) receptors, while the F fusion glycoprotein drives membrane fusion and cytoplasmic release of the ribonucleoprotein complex. EFNB2 is widely expressed in the arterial endothelium and peripheral tissues [94], whereas EFNB3 is enriched in the CNS, explaining the virus’s dual endothelial and neuronal tropism [95]. Following membrane fusion, viral RNA serves as a template for transcription and replication, orchestrated by the polymerase complex composed of P and L proteins. A characteristic transcriptional gradient ensures preferential expression of genes located at the 3′ end of the genome [93].

Once primary replication in the epithelial cells is established, NiV spreads to the vascular endothelial cells, triggering syncytium formation, endothelial inflammation, and vasculitis (Figure 4, steps 5–6). This endothelial damage disrupts the vascular integrity, allowing the virus to enter the bloodstream and disseminate systemically. Hematogenous dissemination results in infection of multiple organs, including the lungs, heart, kidneys, and spleen, potentially leading to multi-organ dysfunction syndrome. In addition, by binding to heparan sulfate, the virus attaches itself to circulating leukocytes without actually infecting them. In this way, NiV exploits white blood cells as transport vehicles to spread throughout the host organism [96]. NiV ultimately reaches the central nervous system, where it induces encephalitis associated with both vascular damage and neuronal infection. This process is facilitated by disruption of the blood–brain barrier (BBB) following infection of cerebral microvascular endothelial cells (Figure 4 9a,9b). In addition, based on experimental studies in hamsters, viral entry into the central nervous system is proposed to occur via transneuronal spread from the olfactory epithelium through the cribriform plate to the olfactory bulb, enabling early CNS access prior to the onset of neurological signs [97] (Figure 2).

Histopathological analyses in fatal human cases have confirmed widespread vasculitis, thrombosis, and parenchymal necrosis, with viral antigens predominantly detected in the lungs, kidneys and brain [7]. It is worth noting that, in humans, the virus seems to spare the liver and skeletal muscles from any pathological damage [98].

Experimental studies on cats have shown that infection with NiV, whether administered subcutaneously or via intranasal or oral routes, can lead to broncho-interstitial pneumonia as well as signs of meningitis [99]. Highly pathogenic and acute infection models have been developed in nonhuman primates such as squirrel monkeys and African green monkeys, offering insights into disease progression in systems closely resembling humans. These studies have also indicated that persistent infection may result in delayed-onset encephalitis with possible relapses [45]. Pteropid bats, which serve as the natural reservoir of NiV, did not develop apparent clinical symptoms under experimental infection [100]. In contrast, pigs acted as important intermediate and amplifying hosts during outbreaks, exhibiting both respiratory and neurological manifestations accompanied by a fever [101]. Overall, these well-established animal models have significantly contributed to understanding NiV pathogenesis.

### 5.2. Immune Mechanisms and Evasion

Nipah virus (NiV) infection triggers a complex interplay between host pattern recognition pathways and viral countermeasures. Viral RNA is detected by cytoplasmic pattern recognition receptors (PRRs) such as RIG-I (retinoic acid-inducible gene I) and MDA5 (melanoma differentiation-associated protein 5), which ordinarily initiate type I interferon (IFN) signaling and downstream antiviral gene expression [93]. However, NiV encodes multiple P gene products—including V, W, and C proteins—that antagonize key PRR signaling cascades, interfering with STAT1 and STAT2 (signal transducer and activator of transcription 1 and 2) nuclear translocation and suppressing interferon-stimulated gene (ISG) activation [93,102]. In addition to these canonical evasion strategies, emerging evidence indicates that NiV may modulate other PRR pathways, such as Toll-like receptors (TLRs), further reshaping the balance between protective immunity and pathogenic inflammation.

Despite viral countermeasures, NiV infection induces robust inflammatory responses characterized by production of cytokines and chemokines—including TNF-α (tumor necrosis factor alpha), IL-6 (interleukin 6), IL-8 (interleukin 8), MCP-1/CCL2 (monocyte chemoattractant protein 1/C-C motif ligand 2), and CXCL10 (C-X-C motif chemokine ligand 10)—which promote leukocyte recruitment, endothelial activation, and an increased vascular permeability [103]. Within the central nervous system (CNS), endothelial and glial cell responses contribute both to containment of infection and to immunopathology. Astrocytes, which express ephrin-B2 and support productive viral replication, undergo reactive astrogliosis and release chemokines that facilitate immune cell infiltration across a compromised blood–brain barrier [104].

From a translational perspective, these insights highlight potential host-directed therapeutic targets, aimed at restoring interferon responsiveness or modulating dysregulated cytokine production, potentially complementing direct-acting antivirals and neutralizing monoclonal antibodies in mitigating disease progression. Understanding the interplay between viral evasion strategies and host defenses remains essential for the design of next-generation countermeasures against NiV infection.

### 5.3. Nipah Virus Encephalitis

Encephalitis represents the hallmark and most severe manifestation of NiV infection and is a major determinant of the clinical outcome [105]. Histopathological features of NiV encephalitis are characterized by the combined contribution of systemic vasculitis with microinfarction and direct neuronal infection, with experimental evidence suggesting that neuronal involvement plays a predominant role in acute disease [106]. Once within the CNS, NiV exhibits a marked tropism for neurons, which represent the primary cellular target of infection. In experimentally infected African green monkeys, widespread viral dissemination has been observed across multiple brain regions, including the brainstem, cerebral cortex, and cerebellum, with the viral antigen predominantly localized in neurons and, in surviving animals, also in microglial cells [107]. Mechanisms of viral persistence within the CNS are not fully understood, but are supported by evidence of a prolonged viral presence in neuronal and glial compartments in animal models [107]. A recent study [106] that analyzed brain tissues (cerebrum, brainstem, and cerebellum) obtained from 15 autopsy cases identified three distinct categories of parenchymal lesions: (i) areas of necrosis lacking detectable viral antigens; (ii) regions with neuroglial immunohistochemical (IHC) positivity for viral antigens in the absence of, or with only minimal, necrosis; and (iii) regions showing both neuroglial immunoreactivity for viral antigens and associated necrosis. These investigations yielded several key observations. The majority of cells positive for viral antigens or RNA were neurons, suggesting that direct neuronal infection represents a primary driver of pathogenesis in acute encephalitis, rather than microinfarction.

Astrocytes, abundant glial cells of the central nervous system that maintain neuronal homeostasis and blood–brain barrier integrity, play a dual role in Nipah virus encephalitis, serving both as targets for infection and as amplifiers of neuroinflammation. These cells express high levels of ephrin-B2 and support productive viral replication, contributing to viral spread through syncytium formation [104].

Infected astrocytes undergo reactive astrogliosis, characterized by hypertrophy, increased GFAP (glial fibrillary acidic protein) expression, and release of chemokines such as CXCL10 and CCL5. This response promotes immune cell recruitment across a compromised blood–brain barrier, exacerbating tissue damage. Astrocyte dysfunction further impairs glutamate clearance, leading to excitotoxic neuronal death, cerebral oedema, and seizures [104].

Neuroimaging findings, while not directly part of pathogenesis, are consistent with the underlying neuropathology and typically show multiple small (2–7 mm) lesions distributed in the subcortical and deep white matter of the cerebral hemispheres [107].

### 5.4. Clinical Manifestations

The clinical presentation of Nipah virus (NiV) infection is highly variable, but it is typically associated with severe disease and a high mortality. The incubation period ranges from a few days up to two months, most commonly around 5–10 days [108]. In the early phase, patients usually develop nonspecific, flu-like symptoms such as a fever, a headache, myalgia, dizziness, nausea, and vomiting, which can make early diagnosis challenging due to overlap with other common infections [109]. A proportion of infected individuals may remain asymptomatic during this stage [110].

As the disease progresses, patients frequently develop involvement of the respiratory and/or central nervous systems. Respiratory manifestations range from mild symptoms to severe pneumonia and acute respiratory distress syndrome (ARDS), in some cases requiring mechanical ventilation [4,111]. Neurological involvement is a hallmark of NiV infection and often presents as encephalitis, which may occur in acute, subacute, or delayed forms. Common neurological features include an altered consciousness, confusion, drowsiness, seizures, focal neurological deficits, and coma [96,112]. In more severe cases, signs of brainstem dysfunction and motor impairment may also be observed.

Importantly, relapsing or late-onset encephalitis has been described, sometimes occurring months or even years after the initial infection, with clinical features similar to acute encephalitis [111,112]. Differences in clinical presentation have been reported between viral strains: infections caused by the Bangladesh strain (NiV-B) are more frequently associated with prominent respiratory involvement and rapid disease progression, whereas the Malaysian strain (NiV-M) more commonly presents with predominant neurological manifestations [68,113].

In addition to neurological and respiratory disease, NiV infection can involve other organ systems, leading to complications such as myocarditis, pancreatitis, and renal dysfunction [114]. Among survivors, long-term neurological sequelae are relatively common, particularly in those who experienced encephalitis, and may include epilepsy, personality changes, and persistent functional impairment [113,115].

## 6. Therapy

Nipah virus (NiV) encephalitis remains a highly severe disease with a substantial mortality, and as of January 2026, no specific antiviral therapies or licensed human vaccines have been approved [79]. Clinical management is therefore primarily supportive, with investigational agents administered under research protocols or compassionate-use frameworks during outbreaks [20,116]. Among pathogen-directed experimental strategies, neutralizing monoclonal antibodies (mAbs) currently represent the most advanced therapeutic approach, given their ability to block viral entry and potentially limit early systemic dissemination.

In parallel, several antiviral compounds targeting distinct stages of the viral replication cycle are under preclinical and early clinical evaluation, although robust efficacy data in humans remain limited.

### 6.1. Monoclonal Antibodies

NiV entry into host cells is mediated by coordinated activity of the attachment glycoprotein G and the fusion glycoprotein F. Neutralizing mAbs targeting either of these surface proteins have demonstrated a protective efficacy in several animal models, including hamsters, ferrets, and non-human primates [117,118,119]. By preventing receptor engagement or membrane fusion, these antibodies reduce viral replication and systemic spread. In addition to direct neutralization, Fc-mediated effector mechanisms may contribute to antiviral activity, although their relative importance in vivo remains incompletely defined [120].

#### 6.1.1. m102.4

m102.4 is a fully human monoclonal antibody directed against the receptor-binding site of the NiV G glycoprotein [20,117]. Structural and functional studies have shown that it binds with a high affinity to the ephrin-B2/B3 interaction interface, thereby sterically blocking viral attachment to host cells [93,118]. By preventing receptor engagement, m102.4 inhibits the subsequent triggering of the F protein required for membrane fusion, effectively interrupting infection at an early stage.

Preclinical studies have demonstrated a substantial protective efficacy. In ferret and African green monkey (AGM) models challenged with NiV-Malaysia (NiV-M), administration of m102.4 in single- or two-dose regimens conferred survival even when the treatment was initiated up to five days after infection [93,119]. However, in AGMs challenged with the Bangladesh strain (NiV-B), the therapeutic window was narrower, with survival primarily observed when the treatment was initiated within three days post-infection [93,116]. These strain-dependent differences underscore the importance of cautious extrapolation across outbreak settings.

Human data are limited to safety and pharmacokinetic studies. A Phase 1 randomized controlled trial in healthy adults reported no serious adverse events and no detectable anti-drug antibodies [116,120]. m102.4 has also been administered on compassionate grounds for high-risk exposures to Hendra virus in Australia and during Nipah outbreaks in India. No treated individuals developed confirmed disease; however, given the absence of controlled efficacy data, a causal protective effect cannot be definitively established [116]. At present, m102.4 is therefore best considered a promising candidate for post-exposure prophylaxis or early therapeutic intervention, pending further clinical evaluation.

#### 6.1.2. MBP1F5 (1F5)

MBP1F5 (also referred to as 1F5 in preclinical studies) is a humanized monoclonal antibody targeting the prefusion conformation of the NiV F glycoprotein. Unlike m102.4, which blocks viral attachment, 1F5 acts at the membrane fusion stage. Structural analyses indicate that it binds to a critical epitope near the apex of the F trimer, stabilizing the prefusion state and preventing the conformational rearrangements required for fusion of the viral and host membranes [93]. Because the F protein is highly conserved across known NiV lineages, this mechanism is expected to confer a broad neutralizing potential [119].

In comparative AGM studies using NiV-B challenge models, 1F5 administered five days after infection provided complete protection from lethal disease at doses of 25 mg/kg and 10 mg/kg, whereas m102.4 at 25 mg/kg conferred survival in only a minority of treated animals under similar experimental conditions [93,116,121]. These findings suggest a longer therapeutic window in this specific model. Nevertheless, these results are derived exclusively from controlled animal studies, and no human efficacy data are currently available.

As of 2025, early-phase clinical evaluation of anti-F antibodies has been initiated to assess the safety and pharmacokinetics in humans [116,119]. Whether the extended therapeutic window observed in non-human primates will translate into a clinically meaningful benefit in outbreak settings remains to be determined.

#### 6.1.3. Clinical and Pathophysiological Considerations

NiV encephalitis is characterized by both direct neuronal infection and widespread vasculitic involvement of the cerebral microvasculature. By reducing the circulating viral load and limiting infection of endothelial cells, neutralizing mAbs may decrease viral neuroinvasion and attenuate downstream neuroinflammatory responses [117,122]. The timing of administration appears critical: in animal models, the efficacy declines as the infection progresses and systemic and neurological involvement become established.

Current prioritization frameworks identify m102.4 and 1F5 among the most advanced candidates for further development, particularly for post-exposure prophylaxis and early treatment strategies [116]. However, robust Phase 2 efficacy trials, pharmacokinetic–pharmacodynamic optimization studies, and standardized outbreak-ready clinical protocols remain essential before these agents can be considered part of an established standard of care.

In summary, monoclonal antibodies targeting NiV glycoproteins represent the most promising disease-modifying approach currently under investigation. Nonetheless, their clinical role in human Nipah encephalitis remains to be definitively defined.

### 6.2. Antivirals

Despite more than two decades of research, no antiviral drugs have yet been approved specifically for Nipah virus infection. The therapeutic landscape has evolved from empirical repurposing during outbreaks to more rational, mechanism-based antiviral development. However, most available data remain preclinical, and the translation from animal models to human disease is still unresolved. Unlike monoclonal antibodies, which prevent viral entry at the extracellular level, small-molecule antivirals act intracellularly, targeting viral RNA synthesis and limiting the production of progeny virions. Their efficacy therefore depends not only on antiviral potency, but also on timing, tissue penetration, and the stage of disease at intervention.

#### 6.2.1. Ribavirin: Historical Use and Persistent Uncertainty

Ribavirin was the earliest antiviral administered during NiV outbreaks and remains the most controversial. It is a broad-spectrum guanosine analog that interferes with viral RNA synthesis and has long been used for hepatitis C and viral hemorrhagic fevers [18]. Because of its availability and prior clinical experience, it was empirically deployed during the 1998–1999 Malaysian outbreak and later during outbreaks in India [123].

During the Malaysian outbreak, ribavirin was administered to 140 patients, with 45 deaths (32%) compared to 29 deaths (54%) in 52 untreated controls, suggesting a 36% reduction in mortality [93,123]. However, allocation was not randomized, and differences in supportive care may have confounded the observed benefit [124]. Subsequent experience has not definitively clarified this ambiguity. In Kerala (2018), ribavirin was used empirically in a small cohort, but no statistically significant reduction in the case fatality rate could be demonstrated [108]. Case reports show heterogeneous outcomes, ranging from survival in isolated cases to uniform mortality despite treatment [125,126].

Mechanistically, ribavirin demonstrates in vitro inhibition of NiV and Hendra virus replication [18,93]. Yet, the in vivo data are less reassuring. In hamster and non-human primate models, ribavirin delayed the time to death, but failed to prevent mortality [127,128,129]. Limited penetration of the blood–brain barrier may explain this discrepancy: while viral titers in the respiratory tract may be reduced, the CNS infection proceeds unchecked [127]. Moreover, ribavirin is associated with teratogenicity and significant adverse effects with prolonged administration [18].

Taken together, ribavirin represents an important historical attempt at antiviral intervention, but cannot currently be considered a reliable or evidence-based standard of care.

#### 6.2.2. Remdesivir: Strong Preclinical Signal, Narrow Window

Remdesivir has emerged as the most compelling small-molecule candidate based on non-human primate data. It is a monophosphoramidate prodrug of an adenosine nucleotide analog that inhibits the viral RNA-dependent RNA polymerase (RdRp) [130]. After intravenous administration, remdesivir undergoes intracellular metabolic activation to its active nucleoside triphosphate metabolite (GS-443902), which competes with adenosine during RNA synthesis. Incorporation into the growing viral RNA strand results in delayed chain termination, stalling the polymerase and halting genome replication [130].

In African green monkeys infected with NiV-B, treatment initiated 24 h post-infection resulted in complete survival [130]. When administration began three days post-infection, protection became dose-dependent and incomplete, with survival rates of ~67% in high-dose groups; the surviving animals displayed histological brain lesions [131]. Timing is therefore critical, and the requirement for intravenous administration further constrains its use in large or resource-limited outbreaks. Additional post-exposure data support its efficacy in non-human primates [132].

#### 6.2.3. Favipiravir: Operational Advantages, Translational Uncertainty

Favipiravir (T-705) is a purine analog prodrug converted intracellularly into its active ribofuranosyl-5′-triphosphate metabolite; it inhibits viral RdRp and can induce lethal mutagenesis [133,134]. In vitro, favipiravir inhibits NiV and Hendra virus replication at micromolar concentrations [133]. In Syrian hamster models, immediate post-exposure administration resulted in complete protection against a lethal NiV challenge [133]. However, most data involve the Malaysian strain (NiV-M); protection against the Bangladesh strain (NiV-B) has not yet been conclusively demonstrated. Favipiravir has undergone clinical trials and is approved in Japan for influenza [18], but no direct clinical data currently support its use in NiV infection.

#### 6.2.4. Toward Multi-Target Therapeutic Strategies

The partial efficacy of ribavirin, the time-sensitive protection conferred by remdesivir, and the strain-dependent uncertainty surrounding favipiravir suggest that monotherapy may be insufficient for advanced disease. NiV pathogenesis involves both extracellular viral dissemination and intracellular replication, processes that may require complementary therapeutic targeting. Preclinical evidence therefore supports a dual-target rationale combining extracellular neutralization of viral entry with intracellular suppression of replication.

##### DS90–m102.4: A Dual-Targeting Bispecific Immunotherapy

DS90–m102.4 has been engineered as a bispecific antibody integrating two distinct neutralizing specificities within a single molecular construct [135]. It combines:

- m102.4, a human monoclonal antibody targeting the receptor-binding site of the RBP (G) glycoprotein;
- DS90, a camelid-derived single-domain nanobody against a conserved prefusion epitope of the F fusion protein.

The construct blocks RBP-ephrin receptor interaction and stabilizes F in its prefusion conformation, inhibiting membrane fusion. In vitro, the bispecific format limits escape mutants compared with individual antibodies, and in a Syrian hamster model of a lethal NiV challenge, DS90–m102.4 demonstrated a superior protection relative to monovalent approaches [118,135].

#### 6.2.5. Concluding Perspective

Antiviral development for NiV has progressed from empirical repurposing to structurally informed, mechanism-based design. Polymerase inhibitors provide important preclinical signals, but are highly time-dependent and may be insufficient once neuroinvasion is established. Emerging dual-target immunotherapies address these limitations by simultaneously blocking viral entry and limiting viral escape. Systematic reviews highlight the need for prioritized clinical evaluation of combination strategies [116]. Whether such strategies will translate into effective clinical interventions remains to be determined.

### 6.3. Vaccines: Current Strategies and Developmental Landscape

Until recently, no vaccines were available for human use against Nipah virus (NiV). However, several candidates have now progressed into early-phase clinical development [136], supported by international initiatives, including the Coalition for Epidemic Preparedness Innovations (CEPI) and the National Institute of Allergy and Infectious Diseases (NIAID) [137,138]. These coordinated efforts reflect the recognition of NiV as a priority pathogen with epidemic potential and high case fatality rates.

Most advanced NiV vaccine candidates are based on established vaccine platforms—adaptable technological systems such as viral vectors, mRNA constructs, or recombinant protein subunits—that can be reconfigured through insertion or expression of pathogen-specific antigens. Across platforms, the principal immunological targets are the viral surface glycoproteins G (attachment protein) and F (fusion protein), which mediate host cell entry and represent the primary antigens capable of eliciting protective immune responses [139].

To illustrate the main technological strategies currently under evaluation, three representative vaccine platforms are examined below: a chimpanzee adenoviral vector-based vaccine (ChAdOx1 NipahB), an mRNA-based vaccine (mRNA-1215), and a recombinant protein subunit vaccine (HeV-sG). These candidates were selected based on their progression into clinical development and the availability of comparative preclinical and immunogenicity data.

#### 6.3.1. ChAdOx1 NipahB (University of Oxford)

The ChAdOx1 (Chimpanzee Adenovirus Oxford 1) Nipah B candidate employs a replication-deficient chimpanzee adenoviral vector. The use of a non-human adenovirus backbone is intended to minimize interference from pre-existing anti-vector immunity in human populations. The “B” designation refers to the Bangladesh strain of NiV, which has been associated with increased transmissibility in human outbreaks.

Following intramuscular administration, the recombinant adenoviral vector enters host cells and delivers a DNA sequence encoding the NiV G glycoprotein. Although replication-incompetent, the vector mediates transient intracellular expression of the G antigen, which is subsequently processed and presented to the immune system, leading to both humoral and cellular immune responses.

A preclinical evaluation in African green monkey (AGM) models demonstrated induction of NiV-G-specific IgG and neutralizing antibody responses after both single- and two-dose regimens [140]. Vaccinated animals were protected against a lethal NiV-B challenge, with evidence of near-sterilizing immunity, defined as minimal or undetectable viral replication following the challenge [139]. These findings support the role of G-directed immune responses in blocking viral attachment to ephrin-B2/B3 receptors and preventing productive infection.

The ongoing Phase I clinical trial (ISRCTN87634044) is evaluating safety and immunogenicity in healthy adults using single- and two-dose schedules [141]. Early clinical data indicate an acceptable tolerability and induction of antigen-specific immune responses [136].

A key challenge in NiV vaccine development is the identification of a reliable immunological correlate of protection. Although neutralizing antibodies are generally considered central to protection against paramyxoviruses, experimental data suggest that protection against NiV may not rely exclusively on high neutralizing titers. In certain non-human primate challenge studies, survival was observed despite low or undetectable neutralizing antibody levels at the time of exposure [142,143,144]. These findings indicate that additional mechanisms, including T-cell-mediated immunity and Fc-dependent antibody effector functions (e.g., antibody-dependent cellular cytotoxicity and complement activation), may contribute substantially to protective immunity.

#### 6.3.2. mRNA Vaccine (Moderna–mRNA-1215)

The mRNA-1215 vaccine, developed by Moderna in collaboration with the Vaccine Research Center and NIAID, represents a lipid nanoparticle-formulated mRNA platform [145,146,147]. The numeric designation “1215” corresponds to an internal development identifier.

This candidate encodes a chimeric antigen incorporating a prefusion-stabilized NiV F glycoprotein covalently linked to G protein components [145]. Stabilization of the F protein in its prefusion conformation is intended to preserve conformational epitopes associated with potent neutralizing activity. The mRNA construct incorporates N1-methyl-pseudouridine to enhance the molecular stability and translational efficiency, and the coding sequence undergoes optimization to improve antigen expression in human cells [145,147].

Preclinical studies demonstrated induction of robust neutralizing antibody responses against both the NiV-Malaysia and NiV-Bangladesh strains, as well as cross-reactivity with Hendra virus. In addition to humoral responses, antigen-specific cellular immune responses were detected [146], supporting the immunogenic potential of the chimeric construct.

A Phase I dose-escalation clinical trial (NCT05398796) evaluated the safety, tolerability, and immunogenicity in healthy adult volunteers [148]. The dose-escalation design permits systematic assessment of increasing antigen doses to determine an optimal balance between immunogenicity and safety. From a translational perspective, the mRNA platform offers a rapid antigen redesign capability and scalable manufacturing processes, attributes that are particularly relevant for outbreak preparedness.

#### 6.3.3. HeV-sG Recombinant Subunit Vaccine

The HeV-sG vaccine is a recombinant protein subunit candidate based on the soluble (s) G glycoprotein of Hendra virus. The antigen is produced using recombinant DNA technology, whereby the gene encoding the HeV G protein is expressed in cultured cells and subsequently purified [149].

Removal of the transmembrane domain generates a soluble form that facilitates secretion and purification. The purified protein is formulated with adjuvants such as aluminum hydroxide and CpG oligonucleotides, which enhance innate immune activation through stimulation of receptors such as Toll-like receptor 9, thereby promoting adaptive immune responses [150].

The rationale for this approach is based on the approximately 83% amino acid homology between HeV and NiV G glycoproteins [151]. Immunization with HeV-sG induces cross-neutralizing antibodies capable of neutralizing both Hendra and Nipah viruses [104].

In non-human primate models, a single-dose regimen conferred complete protection against a lethal NiV challenge, including protection against both the NiV-Malaysia and NiV-Bangladesh strains [152]. Phase I clinical data indicate that two-dose regimens administered 28 days apart elicit substantially higher neutralizing antibody titers than a single-dose administration [153].

#### 6.3.4. Limitations to Phase III Efficacy Evaluation

Despite an encouraging preclinical efficacy and favorable Phase I immunogenicity profiles, no NiV vaccine candidates have progressed to Phase III efficacy trials. The principal limitation is epidemiological: NiV outbreaks are sporadic, geographically restricted, and characterized by relatively low annual case numbers.

Modeling analyses indicate that, under the current incidence patterns in endemic regions such as Bangladesh, conventional randomized Phase III trials using clinical disease endpoints would require extended durations to achieve an adequate statistical power [154]. Consequently, traditional efficacy trial designs may not be feasible.

Alternative regulatory strategies have therefore been proposed, including reliance on validated animal challenge models and the identification of immunological correlates of protection to support licensure under alternative approval pathways [154,155,156]. The development and validation of such surrogate endpoints will be critical for advancing NiV vaccines toward regulatory approval and practical deployment. For an overview of the current therapeutic and vaccine strategies against Nipah virus infection, refer to Table 2.

## 7. Disease Risk and Management

Beyond the therapeutic and vaccine strategies summarized in Table 2, effective public health management is crucial to reduce Nipah virus transmission. The experience from the Kerala outbreaks provides valuable lessons in outbreak containment, risk mitigation, and a One Health approach, which can inform global preparedness strategies.

Nipah virus outbreaks are driven by multiple factors, including viral genetic variability, human–animal interactions, and environmental conditions. Effective risk management relies on early detection, rapid isolation of cases, and contact tracing, alongside public awareness campaigns to reduce exposure to potentially contaminated food sources such as date palm sap or infected animals [157].

The Kerala model exemplifies successful containment measures, emphasizing how outbreak control can be strengthened through a series of coordinated actions [8,158]. In particular, it highlights (i) the rapid establishment of dedicated isolation facilities combined with the implementation of high-level infection control practices in hospitals, ensuring prompt containment of suspected and confirmed cases; (ii) the integration of multidisciplinary teams, bringing together clinicians, epidemiologists, veterinarians, and local public health officials to enable a comprehensive and coordinated response; (iii) the active engagement of communities through education programs aimed at increasing public awareness of transmission risks and promoting preventive behaviors; and (iv) close coordination with wildlife and environmental authorities to monitor potential spillover events from bats, recognized as the natural reservoir of NiV.

This approach highlights the importance of a One Health framework, which integrates human, animal, and environmental health perspectives. Such coordinated measures, when implemented alongside surveillance systems and outbreak preparedness protocols, can significantly reduce transmission and mortality during NiV outbreaks [8,158].

Future directions should include strengthening regional and global surveillance, rapid diagnostic capabilities, and modeling of high-risk zones to preemptively target interventions. The lessons learned from Kerala can serve as a model for other regions with potential NiV exposure, demonstrating how structured public health interventions complement vaccination and antiviral strategies.

## 8. Conclusions

Nipah virus (NiV) encephalitis remains one of the most severe emerging zoonotic diseases, characterized by high case fatality rates, rapid neurological decline, and complex multisystem involvement. The pathogenic mechanisms underlying NiV disease reflect a coordinated interplay between viral determinants and host responses. Viral entry via ephrin-B2 and ephrin-B3 receptors, endothelial tropism, disruption of the blood–brain barrier, and suppression of type I interferon signaling collectively contribute to systemic vasculitis, neuronal infection, and the development of acute and relapsing encephalitis. Continued investigation of these molecular and cellular mechanisms remains essential to inform rational countermeasure design.

Therapeutic development has progressed substantially in recent years. Neutralizing monoclonal antibodies targeting the G and F glycoproteins represent the most advanced pathogen-specific strategy, demonstrating protection in non-human primate models. Small-molecule antivirals targeting viral replication provide complementary approaches, although their clinical utility may be limited by narrow therapeutic windows and challenges in central nervous system penetration. The available evidence suggests that early intervention and potentially combination-based strategies may be necessary to optimize the clinical outcomes.

Vaccine development has advanced to early-phase clinical trials using viral vector, mRNA, and recombinant protein subunit platforms. These technologies have shown robust immunogenicity and protection in relevant animal models. However, the sporadic and geographically restricted nature of NiV outbreaks complicates traditional efficacy evaluations. Identification of reliable immunological correlates of protection therefore represents a critical step toward regulatory advancement and large-scale deployment.

Beyond biomedical countermeasures, NiV highlights the broader biological and ecological context of emerging zoonoses. Environmental change, expanding human–bat interfaces, and healthcare-associated transmission amplify the outbreak risk. Integrating molecular virology, immunology, ecological surveillance, and translational research within a “One Health framework” will be essential for sustainable preparedness.

In summary, substantial progress has been achieved in understanding NiV neuroinvasion and in developing candidate therapeutics and vaccines. Nevertheless, bridging the gap between experimental advances and deployable public health tools remains a central challenge. Coordinated interdisciplinary efforts will be crucial to mitigate the impact of future Nipah virus outbreaks and to strengthen preparedness against high-consequence emerging pathogens.

## Figures and Tables

**Table 1:** Major documented outbreaks of Nipah virus infection worldwide (1998–2026).

No.	Year	Country	Location	Cases	Deaths	CFR (%)	Main Transmission Pattern	Ref.
------			------------------------	-----------	--------------		-----------------------------	
1	1998–1999	Malaysia	Perak, Negeri Sembilan, Selangor	265	105	39.6	Pig-to-human	[29,67]
2	1999	Singapore	Abattoir workers	11	1	9.1	Imported pigs	[58]
3	2001	India	Siliguri, West Bengal	66	45	68.2	Human-to-human (nosocomial)	[64]
4	2001	Bangladesh	Meherpur	13	9	69.2	Bat-to-human spillover	[68]
5	2003	Bangladesh	Naogaon	12	8	66.7	Bat-to-human spillover	[69]
6	2004	Bangladesh	Rajbari	31	23	74.2	Foodborne (date palm sap)	[70]
7	2004	Bangladesh	Faridpur	36	27	75.0	Human-to-human	[70]
8	2005	Bangladesh	Tangail	12	11	91.7	Foodborne transmission	[56]
9	2007	India	Nadia, West Bengal	5	5	100	Bat-to-human spillover	[71]
10	2007	Bangladesh	Kushtia/Pabna	8	5	62.5	Human-to-human	[72]
11	2008	Bangladesh	Manikganj	11	9	81.8	Bat-to-human spillover	[73]
12	2009	Bangladesh	Rangpur	4	1	25.0	Sporadic spillover	[73]
13	2010	Bangladesh	Faridpur	16	14	87.5	Nosocomial transmission	[74]
14	2011	Bangladesh	Multiple districts	44	40	90.9	Seasonal outbreaks	[55,74]
15	2013	Bangladesh	Multiple districts	24	21	87.5	Recurrent zoonotic spillover	[55]
16	2014	Philippines	Sultan Kudarat	17	9	52.9	Horse-to-human transmission	[59]
17	2018	India	Kozhikode/Malappuram, Kerala	23	17	73.9	Human-to-human	[8]
18	2019	India	Ernakulam, Kerala	1	1	100	Sporadic spillover	[75]
19	2021	India	Kozhikode, Kerala	1	1	100	Bat-to-human spillover	[76]
20	2023	India	Kozhikode, Kerala	6	2	33.3	Bat-to-human and human-to-human	[77]
21	2024–2025	India	Kerala state	Sporadic cases	Sporadic deaths	—	Spillover events	[78]
22	2026	Bangladesh	Naogaon district	1	1	100	Bat-to-human spillover	[79]

**Table 2:** Therapeutic and vaccine strategies for Nipah virus infection.

Intervention	Type	Mechanism	Stage of Administration	Evidence	Efficacy	Limitations
m102.4	mAb	Targets G glycoprotein; blocks ephrin-B2/B3 receptor binding	Early infection; post-exposure prophylaxis	Non-human primates; Phase I	Survival up to 5 days post-infection (Malaysia strain); reduced window in Bangladesh strain [93,116,119]	No controlled human efficacy data
1F5 (MBP1F5)	mAb	Targets prefusion F glycoprotein; inhibits membrane fusion	Early infection	Non-human primates	Complete protection when administered 5 days post-infection (Bangladesh strain) [93,116]	No human data
DS90–m102.4	Bispecific antibody	Dual targeting of G and F glycoproteins	Early infection (experimental)	In vitro; hamster model	Improved protection compared to monotherapy [135]	Early-stage development; no primate or human data
Ribavirin	Antiviral	Guanosine analog; inhibits viral RNA synthesis	Early/systemic infection	Observational human studies; animal models	Approx. 36% reduction in mortality in Malaysian outbreak; inconsistent results in later outbreaks [108,123]	Non-randomized data; limited CNS penetration; toxicity
Remdesivir	Antiviral	Inhibits RNA-dependent RNA polymerase (RdRp)	Early infection	Non-human primates	100% survival when given at 24 h; ~67% survival when started at day 3 [107,131]	Narrow therapeutic window; intravenous administration
Favipiravir (T-705)	Antiviral	Purine analog; inhibits RdRp and induces lethal mutagenesis	Early infection; post-exposure	In vitro; hamster model	Complete protection in hamster model (Malaysia strain) [133]	No human efficacy data; limited data for Bangladesh strain
ChAdOx1 NipahB	Viral vector vaccine	Chimpanzee adenoviral vector expressing NiV G glycoprotein	Pre-exposure (prophylaxis)	Non-human primates; Phase I	Protection against lethal challenge; near-sterilizing immunity [139,140]	No Phase III efficacy data
mRNA-1215	mRNA vaccine	Encodes prefusion-stabilized F and G glycoproteins	Pre-exposure (prophylaxis)	Preclinical; Phase I	Induces neutralizing antibodies against multiple strains [146,147]	No efficacy data
HeV-sG	Recombinant subunit vaccine	Soluble Hendra virus G glycoprotein; induces cross-neutralizing antibodies	Pre-exposure (prophylaxis)	Non-human primates; Phase I	Complete protection in primate models [152,153]	Requires adjuvants; multi-dose regimen

Abbreviations: NiV, Nipah virus; HeV-sG, soluble (s) Hendra virus G glycoprotein; mAb, monoclonal antibody; G, attachment glycoprotein; F, fusion glycoprotein; RdRp, RNA-dependent RNA polymerase; CNS, central nervous system; NHP, non-human primate; NiV-M, Malaysia strain; NiV-B, Bangladesh strain.

## Data Availability

No new data were created or analyzed in this study. Data sharing is not applicable to this article.
